# Elusive Sources for Gender Differences in Spatial Ability: The Role of Personality, Spatial Interests, and Everyday Behaviours

**DOI:** 10.11621/pir.2025.0103

**Published:** 2025-03-01

**Authors:** Elina S. Tsigeman, Ksenia V. Bartseva, Evgeniia A. Alenina, Elena L. Soldatova, Yulia V. Kovas, Maxim V. Likhanov

**Affiliations:** a Saint Petersburg State University, Russia; b HSE University, Moscow, Russia; c Beijing Normal University, China

**Keywords:** Big Five, personality, Dark Triad, spatial ability, spatial interest

## Abstract

**Background:**

After decades of research, gender differences in spatial abilities (SA) remain poorly understood. Among factors that may contribute to these differences are self-perceived SA, inclinations, everyday behaviour and interests in SA-related activities, and related personality characteristics. In order to understand these links, a multifactorial approach is needed.

**Objective:**

This study explored the relationships among SA, spatial interests, and personality among adolescent boys and girls.

**Design:**

The study recruited 660 participants (mean age = 15.04, SD = 1.08; 48 females) from public schools. Participants contributed data on a battery of SA tests; 8 personality traits: Big Five and Dark Triad; as well as SA-related activities: spatial interests and school commute information (mode and time) as a proxy for everyday spatial behaviour.

**Results:**

Weak-to-moderate mean gender differences were observed: males scored higher on spatial ability, spatial interests, machiavellianism, and psychopathy; and females on agreeableness, openness to experience, and neuroticism. Hierarchical regression analyses revealed some gender differences in associations among personality traits and SA. In males, openness to experience and conscientiousness were significantly related to SA test performance. In females, openness to experience, extraversion, agreeableness, and machiavellianism contributed to SA performance. Although spatially-related interests were linked to SA, they were not significantly predicted by personality traits. Everyday spatial behaviour showed no relationship with personality traits or SA.

**Conclusion:**

The study replicated patterns of gender differences in spatial ability, spatial interests, and personality reported in previous literature. The results showed differential links between personality traits and spatial ability for males and females. However, the overall amount of variance explained in spatial ability was very small, suggesting that other factors are more prominent sources of gender differences in spatial ability.

## Introduction

Spatial ability (SA) is the fundamental capacity to operate spatial information, and is an important concept in education, especially when it comes to STEM (Science, Technology, Engineering, and Mathematics) areas (Kell et al., 2013). SA has also been studied in the context of largely unexplained gender differences in STEM engagement and success (Stoet & Geary, 2018; Yoon & Mann, 2017). Among factors that may contribute to these differences are interest in SA-related activities and everyday behaviours, as well as personality characteristics. The aim of this study is to provide new insights into gender differences in spatial performance by exploring the relationships among SA, spatial interests, everyday spatial behaviours, and personality traits in males and females.

### Personality-SA Links

Personality traits are defined as persistent patterns of behaviour and emotional reactions (Roberts et al., 2008). Personality traits are related to cognitive performance, for example, in general cognitive ability tests (Carretta & Ree, 2018; Kowalski et al., 2018; Rammstedt et al., 2018; Schaie et al., 2004; Soubelet & Salthouse, 2011; Stanek & Ones, 2023; Voronina et al., 2016); verbal reasoning tasks (Schaie et al., 2004; Sutin et al., 2019), and academic achievement (Chamorro-Premuzic & Furnham, 2003; Meyer et al., 2019). Studies also found associations between personality and both small-scale SA (mental operations with objects) and large-scale SA (processing egocentric spatial information, *e.g.*, during navigation) (Bryant, 1982; Carbone et al., 2019; Meneghetti et al., 2020; Pazzaglia et al., 2018; Stanek & Ones, 2023). For example, participants’ personality traits (capacity for status, sociability, and self-acceptance, etc.) were linked with performance on a large-scale (egocentric) pointing task (Bryant, 1982).

The link between SA and personality could be driven by several mechanisms. For example, according to the investment theory developed by Cattel (Cattell, 1957), personality traits may be related to cognitive performance through motivation and aptitude for learning and exploration. Specifically, individuals high in openness to experience may seek new information more actively (Soubelet & Salthouse, 2011; von Stumm & Ackerman, 2013) and thus have higher cognitive ability. In line with this, two meta-analyses (Stanek & Ones, 2023; von Stumm & Ackerman, 2013) showed that intelligence is linked to personality traits related to intellectual investment (need for cognition, openness to experience, etc.).

With regard to spatial ability, personality traits may contribute to spatial performance via its influence on spatially-related interests, attitudes, self-efficacy, and engagement in spatial activities. In support of this, one study found that the Big Five traits, with the exception of conscientiousness, were associated with attitudes towards spatial exploration (Meneghetti et al., 2020). Another study discovered weak to moderate correlations between all Big Five personality traits and spatial anxiety, pleasure in exploration, and self-efficacy (Pazzaglia et al., 2018). In Bryant (1982), personality traits were linked to the self-reported sense of direction and spatial anxiety of the participants.

A number of studies demonstrated that SA can be improved through various activities and experiences (Uttal et al., 2013), implying that engagement in everyday spatial activities affects SA. Consistent with this, higher levels of extraversion were linked with greater engagement in sports (Steca et al., 2018), which may indirectly enhance some aspects of SA (Habacha et al., 2014; Voyer et al., 2017). Other studies showed associations between personality traits and video games: for example, high extraversion and low neuroticism were associated with preferences for action video games (Braun et al., 2016) — a genre that was experimentally shown to increase SA (Bediou et al., 2018).

A number of studies suggested that individuals’ styles of everyday engagement with the environment might be linked with accuracy of spatial representations (*e.g.*, Bryant, 1982). For example, commuting preferences, *e.g.*, walking or cycling vs. public transport, may indirectly impact everyday spatial experience and performance (Sattler et al., 2023).

Alternatively, the causal direction of these associations could be reversed or reciprocal. According to the PPIK theory (intelligence-as-process, personality, interests, and intelligence-as-knowledge) by Ackerman (1996), there is a reciprocal relationship between cognition, interests, and personality traits in adults. For instance, individuals with superior navigation skills (intelligence-as-process) may be more likely (interests) to actively explore their surroundings (intelligence-as-knowledge), thereby potentially enhancing their abilities. This explanation is supported by some evidence, including links from intelligence to personality (*e.g.*, sociability; Major et al., 2014) and from spatial ability to interests in STEM (Kell et al., 2013).

Furthermore, the link between personality and cognitive ability may reflect personality influence on performance rather than on actual ability. For instance, individuals with high levels of conscientiousness (associated with higher commitment and orderliness) or machiavellianism (associated with strategic thinking and planning) are likely to perform better on complex cognitive tasks, while high levels of neuroticism (*i.e.*, worry and depression) may have a negative impact on performance (Carbone et al., 2019; Carretta & Ree, 2018; Kowalski et al., 2018; Moutafiet al., 2006; Stanek & Ones, 2023). For example, people higher on neuroticism may perform worse under time pressure (Shaw et al., 2020).

### Gender as a Moderator of Personality-SA Links

The links between personality, SA, and spatial interests may be moderated by gender, as gender differences in mean scores are found in personality traits (Likhanov et al., 2021), SA (Tsigeman et al., 2023; Yuan et al., 2019), navigation behaviour (Munroe et al., 1985), and interests (Hofer et al., 2024; Su et al., 2009). For personality, females scored higher on conscientiousness, openness to experience, neuroticism (Likhanov et al., 2021) and “humility” — a tendency to underestimate their ability and performance, *e.g.*, SA (Hofer et al., 2024; Repeykova et al., 2024). Males scored higher on machiavellianism, psychopathy, and narcissism (Likhanov et al., 2021; Luo et al., 2023).

For spatial ability, many studies have found males to have higher performance than females on spatial tests (Likhanov et al., 2023; Tsigeman et al., 2023; Yuan et al., 2019) and higher self-estimates of SA (which were closer to actual performance) (Hofer et al., 2024), as well as more spatial exploration behaviour (Munroe et al., 1985). Previous studies have also demonstrated gender differences in the links between these measures. For instance, one study found that years of formal education and everyday navigation routines had a stronger relation to SA in male than female children (Munroe et al., 1985). Research also suggested that the gender–ability link develops over time. For example, a recent meta-analysis by Lauer and collaborators (2019) demonstrated that the small male advantage in SA in childhood gradually increases by adolescence. It is possible that personality traits and interests contribute to these changes.

The current study explores the relationships among SA, spatial interests, and everyday behaviour and personality traits in adolescent males and females using a multifactorial approach. We examined the associations between spatial ability (measured with 10 tasks) and personality (Big Five and Dark Triad traits), as well as spatially-related interests and commuting behaviour (commuting mode and time to school).

## Methods

### Participants

Six-hundred and sixty adolescents from public schools in Russia participated in the study (314 females; age range 13–17, M^age^ = 15.4, SD = 1.08). Male and female participants were comparable in age (*t* = .398, *p* = .69).

### Procedure

Participants completed a computerised socio-demographic inventory, personality questionnaires (Big Five and Dark Triad), and spatially-related behaviour items, and a battery of 10 SA tests in groups of up to 25 people. Description of each measure, example items, and reliability information are presented in *Table 1*. The testing session lasted approximately 1.5 hours.

**Table 1:** Questionnaires Used in the Study

Construct	Description	Scales	Example Item	Response Options	Validity
Spatial ability	Spatial ability was assessed using 10 tasks from a gamified online battery, “King’s Challenge”, previously adapted to Russian (Esipenko et al., 2018; Likhanov et al., 2018). The total score was calculated as an average percentage of correct answers in the 10 tests and was used to index general SA.	3D to 2D drawing, 2D to 3D drawing, crosssections, Elithorn mazes, mazes, mechanical reasoning, paper folding, pattern assembly, perspective-taking, and shape rotation.	see Supplementary Materials, Table SI, for description of each test; and Rimfeld et al., 2017 for example items		10 tests were validated in an adolescent sample (Budakova et al., 2021) and showed split-half reliabilities from .56 to .86.
Spatially-related interests	1 item, tapping into spatially related interests. Drawn from Study for Mathematically Precocious Youth (Lubinski et al., 2014)	Spatially-related interests	“How often do you tinker with equipment, mechanical devices, gadgets, or participate in games involving construction?”	1 (Never) to 5 (Very frequently).	NA
Everyday spatial behaviour	2 items, tapping into everyday spatial behaviour, namely how students get to school and how long it takes. Drawn from Study for Mathematically Precocious Youth (Lubinski et al., 2014)	Commuting type	“How do you usually get to school?”	bus (1), underground (2), parents give me a ride (3), walk (4), bike (5).	NA
		Commuting time	“How long does it take you to get to school?”.	Up to 10 min (1), 10-30 min (2), 30- 60 min (3).	
Big Five personality traits	The Russian adaptation of The Big Five Inventory (Shchebeten- ko & Wineshtein, 2010) consists of 44 items. Total scores for each	BF: openness	I see myself as someone original, who comes up with new ideas.	1 (strongly disagree) to 5 (strongly agree).	Cronbach’s *a=* .70- 83 for subscales (Likhanov et al., 2021; Mishkevich,
	trait were computed by averaging the scores of corresponding items.	BF: conscientiousness	I see myself as someone who is a reliable worker.		2016)
		BF: extraversion	I see myself as someone who is outgoing; likes to be with people.		
		BF: agreeableness	I see myself as someone who likes to cooperate; goes along with others.		
		BF: neuroticism	I see myself as someone who get nervous easily.	1 (strongly disagree) to 5 (strongly agree).	
Dark Triad	The Russian adaptation of The Short Dark Triad (Egorova et al., 2015) consists of 27 items. The score for each subscale was computed by averaging correspending items	DT: narcissism	People see me as a natural leader.	1 (strongly disagree) to 5 (strongly agree).	Cronbach’s *a=* .65- 71 for subscales (Likhanov et al., 2021)
		DT: psychopathy	Its true that I can be mean to others.		
		DT: machiavellianism	I like to use clever manipulation to get my way.		

### Statistical Approach

All statistical analyses were conducted using Jamovi (Version 1.6, The Jamovi Project, 2021) and R (R Core Team, 2017). All variables were standardised and screened for univariate outliers. Using the threshold of Z = 3.29 (Field, 2005), less than 5 of the sample were identified as univariate outliers and excluded from the analysis. No multivariate outliers were identified using Mahalanobis distance. Skewness and kurtosis of all variables varied within an acceptable range (below the cut-offof 2 recommended by Darren & Mallery, 2010).

## Results

Descriptive statistics for the study variables are presented in *Table S2*.

Participants showed an uneven distribution between the categories of commuting mode and time to school (see *Table 2*). To adjust these categories for the analysis, we combined them into two groups: active mode (Walk and Bike options) and passive mode (Bus, Underground, and “Parents give me a ride”).

**Table 2:** Frequency of Categorical Variables: Commuting Mode and Time to School

	Commuting mode	N	Total
Passive navigation	Bus	107	
	Underground	8	258
	Parents give me a ride	143	
Active navigation	Walk	383	387
	Bike	4	
	**Commuting time**		
	10 min	332	
	10–30 min	252	
	30–60 min	51	

*Table S3* in Supplementary Materials presents the correlations between continuous study variables. All personality traits displayed significant intercorrelations with varying effect sizes. Strong intercorrelations were also observed among all SA tasks. There were negligible-to-weak correlations between all SA facets and some personality traits, namely conscientiousness, extraversion, openness and narcissism. Given largely uniform correlations between different SA facets and personality, we used spatial ability total score for further analysis.

*Table 3* examines personality and spatial ability, interests and behaviour associations using the non-parametric Spearman correlations, as commuting modes are categorical/interval variables. Spatial ability total score correlated with some personality traits and spatial interests. Commuting behaviour showed minor correlations with other variables.

**Table 3:** Non-Parametric Correlation Matrix for Personality Traits, SA, Spatial Interests and Commuting Time

	1	2	3	4	5	6	7	8	9	10
1 Extraversion	–									
2 Agreeableness	.29^***^	–								
3 Conscientiousness	.47^***^	.37^***^	–							
4 Neuroticism	–.46^***^	–.32^***^	–.42^***^	–						
5 Openness	.39^***^	.26^***^	.26^***^	–.15^***^	–					
6 Machiavellianism	–.02	–.34^***^	–.03	.1^*^	.01	–				
7 Narcissism	.55^***^	.03	.27^***^	–.18^***^	.4^***^	.25^***^	–			
8 Psychopathy	–.02	–.54^***^	–.26^***^	.19^***^	–.09^*^	.42^***^	.19^***^	–		
9 Commuting time	.01	–.04	–.08^*^	–.03	.01	.03	.03	.1^*^	–	
10 Spatial ability	–.14^***^	.01	–.14^***^	–.02	.01	.12^**^	–.07	.02	.04	–
11 Spatial interests	.07	.03	.06	–.18^***^	.02	.09^*^	-.02	.06	.06	.17^***^

Note: * p < .05, ** p < .01, *** p < .001

### Hierarchical Regressions: Spatial Ability, Spatial Interests, and Spatial Behaviours

Hierarchical linear regressions were conducted to investigate the unique contribution of personality traits in predicting SA and spatial interests. Additionally, binomial logistic regression was conducted for commuting mode, and multinomial regression was conducted for commuting time. The first step of each model included only age and gender (1 = male; 2 = female) as predictors. In the second step, personality traits were added to the model (see *Table 4* and *Table S4–S5* for all models).

**Table 4:** Hierarchical Regression Analyses with Age, Gender, and Personality Traits as Predictors for Spatial Ability and Spatial Interests

	Spatial ability				Spatial interests			
	Model 1		Model 2		Model 1		Model 2	
Predictors	std. ß	std. CI	std. ß	std. CI	std. ß	std. CI	std. ß	std. CI
Age	.09 *	.02 - .17	.10*	.02-.17	-.08*	-.16- -.01	-.08*	-.16- -.01
Gendera^a^	-.61 ***	-.76 - -.46	-.60 ***	-.76 - -.44	-.70 ***	-.84 - -.55	-.68 ***	-.83 - -.52
Openness			.13**	.04-.21			.08	-.01-.16
Conscientiousness			-.14**	-.23- -.05			.06	-.03 -.15
Extraversion			-.13*	-.23- -.02			.06	-.05 -.16
Agreeableness			.10*	.01-.20			.04	-.06 - .13
Neuroticism			-.04	-.13 - .05			-.05	-.14-.04
Machiavellianism			.11*	.02-.19			.06	-.02 - .15
Narcissism			-.05	-.14-.05			-.12 *	-.22- -.03
Psychopathy			-.02	-.12 - .07			.06	-.04-.15
Observations	642		642		642		642	
R^2^ / R^2^ adjusted	.101 / .099 ***		.154 / .140 ***		.129 / .126 ***		.153 / .140 ***	
DR^2^			.052				.024	
AIC	5608.392		5585.896		2039.184		2037.092	

Note: * p < .05, ** p < .01, *** p < .001; ^a^ Gender was a dichotomous variable (1 = male; 2 = female)

For SA, the predictors explained 15 of the variance. In Step 1, age and gender accounted for 10 of the variance (F (2, 639) = 36.05, *p* < .05), with gender making a larger contribution. In Step 2, personality traits accounted for an additional 5 of the variance (F (10, 631) = 11.46, *p* < .05), with openness, conscientiousness, extraversion, agreeableness, and machiavellianism all making weak significant contributions.

For spatial interests, the predictors collectively accounted for 15 of the variance. Age and gender accounted for 13 of the variance in Step 1 (F (2, 639) = 47.41, *p* < .001), with gender making a larger contribution. In Step 2, personality traits accounted for an additional 2 of significant variance (F (10, 631) = 11.43, *p* < .001), with narcissism being the only trait that made a significant contribution.

For both commuting mode and time, neither model showed statistical significance (see *Tables S4–S5 in the Supplementary Materials*).

### Gender Differences in Predictors of Spatial Ability and Spatial Interests

**Figure 1:**
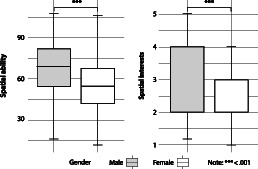
The distribution of SA and spatial interests in male and female participants

First, we assessed gender differences in SA and spatial interests in males and females (see *Table S2*). Figure 1 shows the distribution of spatial interests and SA by gender. Males demonstrated higher SA than females (*t* = 8.09, *p* < .001; Cohen’s *d* = .69) and spatial interests (*t* = 9.37, *p* < .001; Cohen’s *d* = .74).

Additionally, we calculated correlations among personality traits, SA and spatial interests separately in males and females, showing negligible links between personality and SA variables (see Tables S6–S7).

Four separate hierarchical regressions were conducted to investigate the unique contribution of personality traits to SA and spatial interests in males and females (see Tables S8–S9 in Supplementary Materials).

The regression model for males with SA as the outcome showed that openness and conscientiousness significantly contributed to SA, accounting for 5 of the variance (F (9, 326) = 2.151, *p* < .05). The regression model for females showed that openness, extraversion, agreeableness, machiavellianism, and age accounted for 10.5 of the variance in SA (F (9, 296) = 3.875, *p <* .001), with personality traits adding 9 of explained variance. See Table S8 for details. The regression model with spatial interests as the outcome (Table S9) showed that personality had no contribution either for males (F (9, 326) = 1.77, *p =* .07) or for females (F (9, 296) = 1.81, *p =* .06).

## Discussion

The study evaluated links among personality traits, SA, and spatial interests in a large sample of male and female adolescents, to shed new light on gender differences in spatial performance. Our results replicated the results of many previous studies (*e.g.*, Yuan et al., 2019), showing higher spatial ability and spatial interests in males. The study also replicated and extended previous findings of the links between “g” general intelligence and personality — showing links between personality and SA. The results showed some differential links between personality traits and spatial ability for males and females. However, the overall amount of variance explained by personality in spatial ability was very small, suggesting that other factors are more prominent sources of gender differences in spatial ability. Specifically, eight personality traits explained 5 of the variance in SA above age and gender in the overall sample, which is consistent with previous research (Carbone et al., 2019; Schaie et al., 2004). Even less variance (2%) was explained by personality in spatial interests, with only narcissism as a significant predictor beyond age and gender in the overall sample.

The regression analysis in males showed that only openness to experience and conscientiousness significantly predicted SA test performance, together explaining 5 of variance. In contrast, in females more traits contributed to SA test performance: openness to experience, extraversion, agreeableness, machiavellianism, and age explained about 10 of variance. The positive link between SA and openness to experience found in both genders may be explained in the framework of the investment theory (Cattell, 1957): people scoring higher on openness may put more effort into exploring the environment (Rammstedt et al., 2018). Alternatively, people low on openness might be less motivated to explore the environment, and those with lower conscientiousness might be less motivated to complete complex cognitive tasks, thus demonstrating somewhat lower spatial ability.

The links that were identified in either males or females in the current study were previously found in samples that included both males and females. For example, the negative link between cognitive abilities and conscientiousness, found in males, was indicated in previous research (Friedrich & Schütz, 2023; Moutafiet al., 2004). This pattern is in line with the intelligence compensation hypothesis, which suggests that individuals can compensate for lower cognitive ability with higher conscientiousness (Rammstedt et al., 2018). The contribution of machiavellianism to SA, found in females, is in line with previous research and may be due to links between machiavellianism and planning and problem-solving (Carretta & Ree, 2018; Kowalski et al., 2018). The links between SA and extraversion and agreeableness, found in females, were also previously shown in both males and females (Carretta & Ree, 2018; Papageorgiou et al., 2020; Rammstedt et al., 2018; Soubelet & Salthouse, 2011). Furthermore, one recent study has found that openness was associated with positive attitudes toward exploring places in both genders, whereas extraversion was associated with attitudes toward exploring places among men. Higher levels of extraversion were also linked to lower spatial anxiety in both genders, and lower levels of emotional stability were associated with greater spatial anxiety among women (Muffato et al., 2024). Further research is needed to clarify why some of the links were present in males but not females, and vice versa. For example, some of the differences could be explained by differences in absolute levels of these traits — on average higher Big Five personality traits scores in females (Likhanov et al., 2021) and higher spatial ability scores in males (Esipenko et al., 2018; Tsigeman et al., 2023; Yuan et al., 2019).

Males also showed average greater engagement in spatially-related hobbies — consistent with previous studies that found greater engagement in STEM-related hobbies for males (Hofer et al., 2024; Levine et al., 2016). However, correlational analysis did not demonstrate the predicted link between spatial interests, spatial behaviours, and SA (Uttal et al., 2013). We also did not find an association between personality traits and spatial interests in either males or females. The absence of these links may reflect limitations and restrictions that prevent children from acting on their inclinations. For example, their journey to school is often determined by their caregivers and their engagement with hobbies is often limited by availability of resources.

## Limitations and Future Directions

The current study had several limitations:

1. It showed only a small link between spatial ability and spatial interests, presumably because we enumerated a limited number of spatially-related activities that students are engaged in now, in a single item tapping into spatial interests. Future studies in children and adolescents may consider collecting data on desired activities, hobbies, and commuting mode in addition to actual ones, to better understand the links among them and spatial ability (see, for example, some recent studies that explored links between personality, cognitive ability, and engagement with music) (Ruth et al., 2023; Silas et al., 2022). Moreover, we did not consider other activities that might affect spatial ability, and that may account for differences between males and females in spatial abilities, such as sports, videogames or vocational interests (Armstrong et al., 2018; Baerg MacDonald et al., 2023; Kuhn & Wolter, 2022; Tao et al., 2022).
2. The current study utilised a sample of adolescents. Future studies might also consider direct comparisons of the personality–SA links between children/ adolescents and adults, to explore whether these links increase as a result of greater opportunities for adults to act on their inclinations.
3. The current study assessed only small-scale spatial ability in a laboratory setting. Research that documents behaviours in natural settings may shed light on the links between everyday behaviours, such as wayfinding, spatial ability levels, and personality (*e.g.*, one recent study on the links between eye movements during museum exploration and personality traits) (Tsigeman et al., 2024).
4. Longitudinal studies are needed to assess the causal effects of engagement in spatially-related activities on gender differences in spatial ability.
5. The correlational analysis of the links between 10 spatial tasks and 8 personality traits showed a consistent pattern of correlations between personality and spatial ability (*i.e.*, correlations with a specific personality trait was similar regardless of the facet of spatial ability used: there either were correlations with all spatial tasks or no correlations with any task). This suggests little to no specificity in these associations for a specific spatial facet (at least in this sample). Future research will benefit from creating a “general spatial factor”, and loading this factor and residual variance from each spatial task to each individual personality trait, to investigate the specificity of each spatial facet with regards to personality (see a somewhat similar approach in a recent paper that showed specific contributions from a paper-folding task to math word problems, beyond the general intelligence factor; Likhanov etal., 2024).

## Conclusion

Our data demonstrated that the observed gender differences in spatial ability could not be explained by links with personality, spatial interests, and everyday spatial behaviours.

## Supplementary Materials

**Table S1:** Ten Tests of Spatial Ability

Task name	N of items	Time limit per item (sec)	Description	Split-half reliability in Russian sample (M. V. Likhanov et al., 2018)
3D to 2D drawing	5	45	sketching a 2D layout of a 3D object from a specified viewpoint	.78
2D to 3D drawing	7	70	sketching a 3D drawing from a 2D diagram	.80
Cross-sections	15	20	visualizing cross-sections of objects	.74
Elithorn mazes	10	7	joining together as many dots as possible from an array	.88
Mazes	10	25	searching for a way through a 2D maze	.60
Mechanical reasoning	16	25	multiple-choice naive physics questions	.56
Paper folding	15	20	visualizing where the holes are situated after a piece of paper is folded and a hole is punched through it	.85
Pattern assembly	15	20	visually combining pieces of objects together to make a whole	.69
Perspective-taking	15	20	visualizing objects from a different perspective	.86
Shape rotation	15	20	mentally rotating objects	.82

**Table S2:** Descriptive Statistics of Personality Traits, Total Spatial Ability, and Spatial Interests Score for Male and Female Subsamples

Males							Females						
	N	Mean	SD	Min-Max	Skew	Kurtosis	N	Mean	SD	Min-Max	Skew	Kurtosis	t-test
Openness	345	3.67	.62	1.8-5	-.02	-.61	313	3.87	.62	2.1-5	-.28	-.42	-4.18**
Conscientiousness	345	3.63	.65	1.67-5	-.3	-.48	313	3.73	.67	1.89-5	-.21	-.54	-1.84
Extraversion	345	3.58	.76	1.38-5	-.44	-.34	313	3.59	.83	1.12-5	-.38	-.5	-.12
Agreeableness	345	3.69	.54	2.11-5	-.01	-.35	312	3.8	.55	2-5	-.1	-.2	-2.61*
Neuroticism	345	2.57	.74	1-5	.39	.04	313	2.96	.8	1-5	0	-.54	-6.43**
Machiavellianism	345	3.27	.55	1.78-4.89	.03	-.34	313	3.09	.56	1.56-4.56	.04	-.11	4.25**
Narcissism	346	2.99	.55	1.56-4.44	.2	-.2	313	2.98	.54	1.33-4.67	.05	.23	.26
Psychopathy	346	2.18	.48	1.22-3.67	.25	-.28	314	2.02	.5	1-3.56	.45	-.04	4.18**
Total SA score	345	66.74	20.38	16.37-107.46	-.39	-.48	313	54.72	17.72	11.98-106.54	.05	-.46	8.09**
Spatial interests	337	3.38	1.21	1-5	-.33	-.88	309	2.5	1.17	1-106.54	.3	-.95	9.37**

*Note: * p < .01, ** p < .001*

**Table S3:** Correlation Matrix for Age, Personality Domains, Ten Tests Of Spatial Ability, Total Spatial Ability Score, and Spatial Interests

Variable	1	2	3	4	5	6	7	8	9	10	11	12	13	14	15	16	17	18	19
1. Spatial interests	-																		
2. Spatial ability	.17***	-																	
3. Ma	.04	.58***	-																
4. PT		.66***	.26***	-															
5. SR	.11**	.74***	.41***	.39***	-														
6. 3D	.11**	.y4***	.38***	.45***	.49***	-													
7. PF	.07	.75***	.4***	.36***	.45***	.56***	-												
8. MR	.16***	.68***	32***	.39***	.46***	.46***	.43***	-											
9. EM	.17***	.55***	.35***	.33***	.35***	.37***	.34***	.34***	-										
10. PA	.09*	.67***	.38***	.35***	.45***	.4y***	.44***	.35***		-									
11. CS	.12**	.68***	29***	.33***	.41***	.46***	.45***	.47***	.37***	.34***	-								
12. Psychopathy	.06	.03	.04	.01	-.02	-.02	.01	.08*	.07	.01	.06	-							
13. Narcissism	-.01	-.07	.011	-.07	-.09*	-.07	-.06	-.05	-.05	-.07	.02	.19***	-						
14. Machiavellianism	.08*	.11**		.01	.07	.04	.03		.04		.13***	.42***	.25***	-					
15. Openness	.03	.01	.06	-.04	.01	.07	.04	-.03	-.02	-.02	.01	-.07	.4***	.01	-				
16. Neuroticism	- 17***	-.013	.04	-.1*	-.03	-.01	.03	-.03	.01	.01	.02	.21***	- 2***	.12**	-.15***	-			
17. Conscientiousness	.06	-.14***	-.02	12**	-.06	-.14***	-.07	-.11**	-.12**	-.1*	-.13**	- 27***	2y***	-.04	.26***	-.43***	-		
18. Agreeableness	.03	.01	.02	.01	02	.08*	-.02	-.02	-.08	.01	-.03	-.55***	.03	-.35***	.26***	-.33***	.36***	-	
19. Extraversion	.08*	-.13***	-.02	-.1*	-.09*	-.16***	-.12**	-.09*	-.12*	-.07	-.08*	-.02	.57***	-.04	39***	-.47***	.46***	29***	-
20. Age	-.09*	.08*	.09*	.04	.06	.02	.03	.08*	.1*	.01	.11**	-.05	.07	.07	.02	.01	.1*	.03	-.02

*Note: CS — Cross-sections, 2D — 3D to 2D drawing, PA — Pattern assembly, EM — Elithorn mazes, MR — Mechanical reasoning, PF — Paper folding, 3D — 2D to 3D drawing, SR — Shape rotation, PT — Perspective-taking, Ma — Mazes; * p < .05, ** p < .01, *** p < .001 < .001*

**Table S4:** Hierarchical Binomial Logistic Regression with Age, Gender, and Personality Traits as Predictors for Commuting Mode

	Commuting mode ^a^			
	Model 1		Model 2	
*Predictors*	std. β	std. CI	std. β	std. CI
(Intercept)	.65 ^***^	.52– .81	.61 ^***^	.49 – .77
Age	.92	.79 – 1.08	.93	.79 – 1.09
Gender ^b^	1.05	.77 – 1.44	1.13	.80 – 1.61
Openness			1.05	.87– 1.26
Conscientiousness			.90	.74 – 1.10
Extraversion			.91	.72 – 1.14
Agreeableness			1.21	.98 – 1.50
Neuroticism			.90	.73 – 1.10
Machiavellianism			1.03	.86 – 1.25
Narcissism			1.03	.83 – 1.29
Psychopathy			1.23	1.00 – 1.53
Observations	645		641	
R^2^ Tjur	.002		.014	

*Note: a Commuting mode was a dichotomous variable (0 = active mode; 1 = passive mode); b Gender was a dichotomous variable (1 = male; 2 = female)*

**Table S5:** Hierarchical Multinomial Regression Analyses with Age, Gender, and Personality Traits as Predictors for Commuting Time

	Commuting time ^a^			
	Model 1		Model 2	
Predictors	std. β	std. CI	std. β	std. CI
(Intercept) x1	5.63 ^***^	3.81 – 8.32	6.71 ^***^	4.31 – 10.44
(Intercept) x2	4.45 ^***^	2.99 – 6.62	5.08 ^***^	3.24 – 7.96
Age x1	.92	.68 – 1.25	.89	.65 – 1.21
Age x2	.83	.61 – 1.12	.81	.59 – 1.11
Gender ^b^ [Female] x1	1.40	.77 – 2.54	1.11	.57 – 2.14
Gender ^b^ [Female] x2	1.28	.70 – 2.36	1.08	.55 – 2.11
Openness x1			.92	.65 – 1.31
Openness x2			.93	.65 – 1.33
Conscientiousness x1			1.09	.75 – 1.58
Conscientiousness x2			.84	.57 – 1.23
Extraversion x1			1.27	.82 – 1.97
Extraversion x2			1.36	.87 – 2.12
Agreeableness x1			.98	.66 – 1.46
Agreeableness x2			.99	.66 – 1.49
Neuroticism x1			1.40	.95 – 2.08
Neuroticism x2			1.34	.90 – 2.00
Machiavellianism x1			1.17	.83 – 1.65
Machiavellianism x2			1.22	.86 – 1.73
Narcissism x1			.91	.61 – 1.36
Narcissism x2			.99	.66 – 1.49
Psychopathy x1			.64 ^*^	.43 – .96
Psychopathy 2			.68	.46 – 1.02
Observations	635		632	

*Note: a Commuting time was a variable with 3 levels (1 = 10 min; 2 = 10-30 min; 3 = 30-60 min), b Gender was a dichotomous variable (1 = male; 2 = female)*

**Table S6:** Correlation Matrix for Personality Traits, SA, and Spatial Interests in Males

Variable	1	2	3	4	5	6	7	8	9
1. Spatial interests	–								
2. Spatial ability	.14**	–							
3. Psychopathy	.01	–.01	–						
4. Narcissism	.04	–.06	.11*	–					
5. Machiavellianism	.1	.04	.32***	.23***	–				
6. Openness	.11*	.09	–.01	.45***	.07	–			
7. Conscientiousness	.12*	–.09	–.29***	.27***	–.01	.23***	–		
8. Agreeableness	.05	.05	–.52***	.06	–.27***	.25***	.37***	–	
9. Extraversion	.13*	–.09	–.03	.6***	–.05	.41***	.43***	.27***	–
10. Neuroticism	–.07	–.02	.29***	–.14**	.16**	–.14*	–.42***	–.32***	–.44***

*Note: * p < .05, ** p < .01, *** p < .001*

**Table S7:** Correlation Matrix for Personality Traits, SA, and Spatial Interests in Females

Variable	1	2	3	4	5	6	7	8	9
1. Spatial interests	–								
2. Spatial ability	–.01	–							
3. Psychopathy	.01	–.03	–						
4. Narcissism	–.06	–.1	.28***	–					
5. Machiavellianism	–.06	.1	.5***	.27***	–				
6. Openness	.07	.03	–.09	.37***	–.01	–			
7. Conscientiousness	.05	–.16**	–.24***	.28***	–.04	.27***	–		
8. Agreeableness	.08	.01	–.57***	–.01	–.41***	.25***	.34***	–	
9. Extraversion	.05	–.2***	–.02	.55***	–.03	.39***	.5***	.31***	–
10. Neuroticism	–.11*	.17**	.24***	–.26***	.18**	–.26***	–.51***	–.41***	–.52***

*Note: * p < .05, ** p < .01, *** p < .001*

**Table S8:** Hierarchical Regression Analyses with Age and Personality Traits as Predictors for Spatial Ability in Males and Females

	Spatial ability								
	Males				Females				
	Model 1		Model 2		Model 1		Model 2		
Predictors	*std. ß*	*std. CI*	*std. ß*	*std. CI*	*std. ß*	*std. CI*	*std. ß*	*std. CI*	Fisher’s z
Age	.08	-.03-.18	.07	-.04-.18	.12*	.01-.24	.12*	.01-.23	-.636
Openness			.13*	.01-.25			.14*	.02 - .27	-.128
Conscientiousness			-.16*	-.29 - -.03			-.12	-.25 - .02	.514
Extraversion			-.12	-.28 - .04			-.17*	-.32- -.01	-.643
Agreeableness			.09	-.05 - .22			.15*	.01-.30	-.767
Neuroticism			-.12	-.25 - .01			.06	-.08 - .20	.762
Machiavellianism			.08	-.04 - .20			.16*	.02 - .29	-1.023
Narcissism			-.04	-.19-.11			-.05	-.20 - .10	-.126
Psychopathy			-.02	-.15-.12			-.02	-.17-.13	0
Observations	336		336		306		306		
R^2^ / R^2^ adjusted	.006/.003*		.056/.030*		.016/.012*		.105/.078 ***		
DR^2^			.05				.09		
AIC	2977.463		2976.079		2628.843		2615.551		

*Note: * p < .05, ** p < .001*

**Table S9:** Hierarchical Linear Regression Analyses with Age and Personality Traits as Predictors for Spatial Interests in Males and Females

	Spatial ability								
	Males				Females				
	Model 1		Model 2		Model 1		Model 2		
Predictors	*std. ß*	*std. CI*	*std. ß*	*std. CI*	*std. ß*	*std. CI*	*std. ß*	*std. CI*	Fisher’s z
Age	-.04	-.15-.07	-.04	-.15-.07	-.14*	-.26 - -.03	-.11	-.22 - .01	.887
Openness			.08	-.05 - .20			.08	-.04-.21	0
Conscientiousness			.08	-.05-.21			.04	-.10-.18	.506
Extraversion			.14	-.02 - .30			.01	-.15-.17	1.649
Agreeableness			.00	-.13-.14			.07	-.08 - .22	-.883
Neuroticism			-.01	-.14-.12			-.09	-.24 - .05	1.011
Machiavellianism			.14*	.02 - .26			-.03	-.16-,11	2.153*
Narcissism			-.14	-.29-.01			-.16*	-.31 - -.00	.258
Psychopathy			0	-.13-.14			.15	-.01-.31	-1.904*
Observations	336		336		306		306		
R^2^ / R^2^ adjusted	.002/-.001		.047 / .020		.021/.017		.052 / .023		
AIC	1081.569		1082.016		959.387		965.366		

*Note: * p < .05 ** p < .01 *** p < .001*
